# Workplace Attachment Style as Moderator of the Relationship Between Political Skills and Organizational Citizenship Behaviors

**DOI:** 10.5964/ejop.6559

**Published:** 2023-05-31

**Authors:** Jean-Frantz Bruny, Boris Vallée, Fabio Bernardi, Liliane Rioux, Fabrizio Scrima

**Affiliations:** 1 University of Rouen Normandy, Mont-Saint-Aignan, France; 2 Paris Nanterre University, Nanterre, France; 3 Independent Researcher; University of Belgrade, Belgrade, Serbia

**Keywords:** place attachment, workplace attachment style, political skills, organizational citizenship behaviors

## Abstract

A number of studies have demonstrated the role played by political skills on organizational citizenship behaviors (OCBs). Other research has also shown how the work environment can affect OCBs. However, no research has yet addressed the role that workplace attachment style plays in influencing employee OCBs. The present study aims to investigate the moderating role of workplace attachment style on the relationship between political skills and Organizational Citizenship Behaviors (OCBs) using a cross-sectional design. The research was carried out with the participation of 185 French office workers. Research hypotheses were tested by means of three moderation models. The results show that political skills are positively related to OCB, and that secure and preoccupied workplace attachment styles moderate the relationship between political skills and OCB. These results therefore underline the importance of appropriate organizational environmental management in promoting OCBs.

Industrialized countries as a whole have been undergoing major changes from an economic point of view in recent years. Competition is fierce, employees are under great pressure to improve profitability and must be flexible and adapt rapidly to these new working conditions. Understanding employee behavior is therefore of paramount importance to companies, as it has a direct and long-term impact on the performance and health of their employees, as well as on the performance of the company. In this article, we focus on prosocial behaviors in organizational or work environments, that is, discretionary behaviors, “which are not directly or explicitly recognized by a formal reward system and which, on the whole, promote the effective functioning of the organization” (Organ, 1988, p. 4). Numerous studies have highlighted the impact of OCBs on organizational performance (Park, 2018; Podsakoff & MacKenzie, 1997). Organizations are made up of individuals and groups with different values, goals and interests, resulting in potential conflicts over resources (Pfeffer, 1981). Individual characteristics of employees may impact their propensity to activate OCBs (Harper, 2015), including political skills (Ferris et al., 1999a). Here, political skill refers to the social perception and ability to adapt one’s behavior to different and changing situational needs in order to influence others. Political skill is a concept of interpersonal effectiveness that combines social understanding with the ability to adapt behavior to the demands of the situation in a way that appears sincere, and inspires trust and support (Ferris et al., 2005). This study comes within the theoretical framework of the Job Demands-Resources (JD-R) model (Demerouti et al., 2001). In this model, work resources are the characteristics of work that impact the well-being of employees, allow them to achieve goals and develop personal skills (e.g., autonomy) (Bakker & Demerouti, 2007). Studies have already shown that employees manifest more OCB when they receive more job resources (Halbesleben & Wheeler, 2015). Shin and Hur (2019) found that increasing job resources improves daily OCB performance. We suggest that workplace attachment style is a function of the ability of the workplace (job resource) to satisfy the employee’s needs. Within the framework of environmental psychology, the environment is considered to have an effect on employees’ attitudes, behaviors, perceptions and emotions. For example, Turnipseed and Murkison (2000) have shown that physical characteristics of the environment are linked to OCBs, while Le Roy and Rioux (2012) argued that employees who are strongly attached to their workplace are more likely to activate OCBs. Researchers have pointed out that political skills are linked to organizational citizenship behaviors. While the role of political competencies in determining OCBs is clear, the scientific literature has not yet shown how the work environment can positively or negatively influence this relationship. The growing interest of researchers in place attachment has led to an increase in studies related to attachment theory (Bowlby, 1969) in the field of work and organizational psychology, with the emergence of research highlighting the role that attachment styles can play in the interpretation of phenomena and behaviors at work (Richards & Schat, 2011; Scrima et al., 2015). By analogy with Altman and Low’s (1992) definition, workplace attachment can be defined as the emotional bond resulting from the dynamic interaction between individuals and their organizational environment (Rioux, 2006) and is considered an important aspect of quality of work life. In the present research, we were interested in the role played by workplace attachment style in the relationship between political skills and organizational citizenship behaviors (see Figure 1). This study attempts to fill a gap in understanding the changing relational needs of employees and how the satisfaction of these needs can create the potential for organizations to cope with poor OCBs and support employee health, well-being, motivation and satisfaction. It provides much-needed insight into the relationship between workplace attachment styles, political skills, and organizational citizenship behaviors.

**Figure 1:**
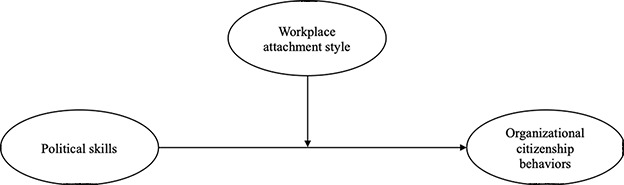
Theoretical Model

## Theoretical Framework

### Organizational Citizenship Behaviors (OCB)

The first observations about citizenship performance were by Barnard (1938), who conceptualized the organization as an association of cooperative efforts. According to this author, employees’ willingness to cooperate is a fundamental aspect of organizational activity, and it is therefore important to reinforce the spontaneous contribution of employees beyond contractual obligations or obedience to legitimate authority. A few years later, Katz (1964) as well as Katz and Kahn (1978) reaffirmed the importance of spontaneity and cooperation for organizational success. Katz (1964) also observed that “an organization that depends solely on prescribed behavior constitutes a very fragile social system” (p. 132). Several researchers have pursued this promising path, giving rise to a plethora of publications on citizenship performance and related constructs, such as organizational citizenship behavior (Bateman & Organ, 1983; Organ, 1988), organizational engagement (Organ & Konovsky, 1989), contextual performance (Borman & Motowidlo, 1997), prosocial organizational behavior (Brief & Motowidlo, 1986), organizational spontaneity (George & Brief, 1992), extra-role behavior (Van Dyne et al., 1995), and mobilization (Tremblay & Wils, 2005). This proliferation of studies is not without consequences and has led to real confusion about the notion of citizenship in organizational settings (Podsakoff et al., 2000). According to Podsakoff et al. (2009), OCB has a positive impact, whether it is directed towards an individual or the organization. Similarly, Lefkowitz (2000) noted that employers like employees who show OCB because they are a model of goodwill in the organization. For this reason, appraising and rewarding employee performance is increasingly based on OCB. However, Dagot and Vonthron (2003) suggested that the effect of OCB goes beyond the framework of in-company appraisal and that it could be a more general indicator of a certain potential to integrate and participate harmoniously in professional teams. Citizen behavior does not lead only to an increase in purely subjective and individual performance but also to an increase in measures of organizational effectiveness, such as productivity, efficiency, profitability and customer satisfaction. It has also been observed to result in reduced intention to leave, lower staff turnover (and therefore fewer related costs) and less absenteeism in companies with a high level of OCB.

### The Relationship Between Political Skills and Organizational Citizenship Behaviors (OCBs)

Although organizational policy has interested researchers for some time, little was known about the skills that individuals use to navigate policy in their workplace until a few decades ago. The term “political competence” was introduced in the scientific literature by Pfeffer (1981) and Mintzberg (1983), who advocated a political perspective of organizations and identified political competence as a requisite for effectiveness in political environments. No further research on political competence was conducted until Ferris and colleagues initiated a research program to understand and measure political competence as a social construct (Ferris et al., 1999a, 2005). Individuals with political competence possess a unique social perception and are able to modify their behaviors in response to different and changing situational needs (Ferris et al., 1999a, 2005). Because people with political skills are able to better understand their colleagues, they can influence others to behave in ways that achieve their own personal or organizational goals. By influencing the behavior of others, politically competent people may feel that they control their colleagues. For people with high levels of political competence, these feelings of self-confidence and control over others reduce feelings of anxiety and work-related stress. Previous research (Perrewé et al., 2000) has shown that highly politically skilled individuals view interpersonal interactions as opportunities, while those with low political skills view them as threats. Individuals with high political skills also interpret environmental stimuli differently than their peers (Ferris et al., 1999a; Perrewé et al., 2000). Munyon and colleagues (2015) extended the meta-theoretical framework established by Ferris et al. (1999a) and discovered, through a meta-analysis, that political skills are positively related to effectiveness, personal satisfaction, job satisfaction, organizational commitment, work productivity, organizational citizenship behavior, professional success and personal reputation, and negatively related to physiological stress. Ferris et al. (1999a) reported that politically competent individuals enjoy personal security and self-confidence in their work environment, resulting in reduced stress and tension at work. Perrewé et al., 2005 found that political skills moderate the relationship between role overload and job strain, job dissatisfaction and general anxiety.

H_1_: Political skills are positively associated with OCB

### The Moderating Role of Workplace Attachment Style

Attachment theory is an established theory of human relationships and is one of the most influential theories in psychology (Cassidy & Shaver, 2002). It has been applied to occupational psychology by many researchers who have highlighted the role that attachment can play in the description and interpretation of phenomena in the work environment (Harms, 2011; Paetzold, 2015; Scrima et al., 2015). Moreover, as environmental psychologists and others have shown, people also develop place attachments (Giuliani, 2003). In general, place attachment is seen as a bond or emotional relationship between people and certain places. Place is a unifying concept because all behavior is associated with a specific place and exists only because that place exists (Rioux, 2006), as defined by many authors. In the most succinct definition, place is a space that has been imbued with meaning through personal, group and cultural processes (Low & Altman, 1992). According to Low and Altman (1992), places thus provide the context in which relationships occur, and people are attached to these social relationships. Attachment to place is a positive and emotional bond that people form with particular places where they feel comfortable and secure and with which they wish to maintain a connection (Hidalgo & Hernandez, 2001; Low, 1992). Recently, some authors (Scannell & Gifford, 2014, 2017) have compared place attachment with the classic theory of attachment (Bowlby, 1969), highlighting many points in common. For example, place attachment provides a sense of security and stimulation because it is based on predictability, routine, and a sense of mastery or control.

Although to date there has been no empirical research on the impact of place attachment styles on various outcomes, the theoretical analysis of the role of place on the attachment process of individuals is recurrent. According to Scrima et al. (2017), the only difference between the process of attachment described by Bowlby and an individual’s attachment to a place is the object of attachment. In Bowlby’s theory, the object of attachment is the caregiver. In the theory of place attachment styles, the object of attachment is the place. Caregivers and specific places share the concept of a safe haven (Bowlby, 1988). According to Bowlby, a caregiver who is able to respond adequately to the child’s requests takes on the function of safe haven. This function, confirmed by the experimental protocol of the Strange Situation (Ainsworth et al., 1978), enables the child to develop autonomy and self-confidence. According to Scannell and Gifford (2013) and Little and Derr (2020), the same function can be ascribed to a specific place such as the home. During childhood, the home ensures the safe haven function, enabling children to develop self-confidence and autonomy (Howe et al., 1999). Autonomy and trust will allow the adult to explore and relate to other places (Morgan, 2010) in a “secure” way. Moreover, Cross (2015) showed that individuals feel place attachment that reflects unique combinations of processes in different places. These findings imply that attachment may survive our current experience, extending into time long after we have left the place. They indicate that attachment to place develops through multiple processes, in multiple places and over time. Thus, home would also play a role in creating internal working models (IWMs) that influence how adults relate to other specific places such as the workplace. Scrima et al. (2017) suggested that individuals develop different workplace attachment styles depending on their representation of self (positive or negative) and of the place (positive or negative). Scrima (2020) developed a scale to measure workplace attachment style. This scale measures a three-dimensional construct and each of the dimensions is used as a separate variable suggesting that it is possible to identify very different behavioral patterns among employees with secure, avoidant or preoccupied attachment styles. Le Roy and Rioux (2012) found a positive relation between workplace attachment and OCBs. They suggested that workplace attachment is a predictor of supportive behaviors. This relationship was later confirmed in studies by Rioux and Pavalache-Ilie (2013) and Pradhan and Mishra (2020). We postulate that employees who perceive that they do not have good political skills but whose needs are satisfactorily met by the workplace environment will develop a secure attachment to the workplace that will foster the probability of activating OCBs. This is in line with other research demonstrating that individuals who feel safe in their workplace show more OCB (Testa et al., 2020); for example, the perception of low levels of polluted air in the workplace could be an indicator of environmental satisfaction in the workplace and lead to the activation of OCBs. Perrewé et al. (2000) observed that employees with high levels of political skills are able to make use of their relational skills. We believe that these employees can use their skills to relate positively to the work environment by developing secure attachment to the workplace. Moreover, Ferris et al. (1999a) found differences in the way that employees with high and low political skills interpret environmental stimuli. In the light of the cited literature, we postulate that a secure workplace attachment style enhances the relationship between political skills and OCBs.

H_2_: Secure workplace attachment positively moderates the relationship between political skills and OCB.

By contrast, if the workplace does not satisfy the needs of employees, they may develop an insecure workplace attachment style. Employees with a preoccupied workplace attachment style have a negative representation of self and a positive representation of place (Scrima et al., 2017). They will therefore feel they are not good enough for the place, which could lead them to activate a series of behaviors to try to reduce proximity (Scannell & Gifford, 2013). The behavioral patterns of individuals with a preoccupied attachment style are determined by the fear of being rejected (Scrima, 2015). The constant search for proximity, the fear of being rejected and feeling of not being up to par could create a constant state of stress. To reduce this state of stress, they may show a high level of commitment to work activities. In order to reduce their stress, employees with a low level of political skills and a preoccupied workplace attachment style may activate OCB. We therefore believe that a preoccupied workplace attachment style reduces the relationship between political skills and OCBs.

H_3_: Preoccupied workplace attachment negatively moderates the relationship between political skills and OCB.

Finally, the avoidant workplace attachment style is characterized by a positive representation of Self but a negative representation of the place (Scrima et al., 2017). This relational style could arise when the object of attachment, which should have been a secure base, does not meet the needs of the individual (Ainsworth et al., 1978), who therefore thinks that he/she does not need the place. Morgan (2010) reported that some participants stated that they had not developed strong attachments to specific places. According to Scannell and Gifford (2013), this could be an indicator of an avoidant place attachment style. The behavioral patterns of the avoidant individual will therefore include poor involvement in activities and a desire for distancing (Martin et al., 2010). This suggests that despite good political skills, a high level of workplace avoidant attachment may reduce the relationship between political skills and OCBs.

H_4_: Avoidant workplace attachment negatively moderates the relationship between political skills and OCB.

## Method

### Sample and Procedure

The sample size for this study was determined using a-priori statistical power analysis (Faul et al., 2007) via G-Power 3.1 software. Conservatively assuming a medium effect size *f*^2^ of .15, with power of .95, alpha of .05, and seven predictors, we calculated a minimum sample size of *N* = 153. The research was carried out with the participation of 185 people working in various organizations in the Île-de-France region. Potential participants were recruited by direct contact on professional websites. To participate in our study, they had to be office workers, to have worked full-time for at least a year in the same organization and to have a permanent contract. This inclusion criterion ensured that the participants had been employed in the same workplace for at least one year, thereby ensuring that they had developed attachment to the workplace (Scrima, 2020). Out of a total of 200 potential participants, 185 completed the questionnaire sent by e-mail (i.e., a response rate of 93%). The study was conducted in line with the American Psychological Association's ethical principles and code of conduct for research with human participants (APA, 2017). Participants were informed about the purpose of the study (i.e., to investigate the relationship between personal characteristics, perception of the workplace and OCBs). No compensation was provided and total anonymity was guaranteed. The questionnaire was divided into three sections: the first contained information about the study’s objectives (i.e., the impact of the workplace on organizational behavior), information about anonymity and a consent form for signature. The second part comprised the scales used in this study. The third part consisted of a series of socio-demographic questions. The sample comprised 65% women and 35% men aged between 21 and 58 years (*M* = 35.07, *SD* = 9.34); 59.5% were office workers, 34.6% were middle managers, and the remaining 5.9% senior managers, with length of service between 1 and 30 years (*M* = 5.43, *SD* = 4.94).

### Measures

The socio-demographic section had questions on sex, age, professional level (1 = office employee, 2 = executiveemployee, 3 = manager), and length of service in years. Political Skills were measured through the French version (Dagot et al., 2014) of the Political Skills Inventory (PSI—Ferris et al., 2005). This scale has 18 items investigating four dimensions of political skills: social intuition, apparent sincerity, interpersonal influence and networking ability (e.g., “I am able to make most people feel comfortable and at ease around me”). It is possible to calculate a score for each dimension and a global score. Participants respond on a 7-point Likert scale (from 1 = totally disagree, to 7 = totally agree). In the present study, we obtained a satisfactory internal consistency index for the overall scale (Alpha = .89). Workplace attachment style was measured using the Workplace Attachment Style Questionnaire (WASQ—Scrima, 2020) (see Appendix). Unlike the dimensional tools more commonly used to measure adult attachment style (Fraley et al., 2000), the WASQ measures three separate dimensions of workplace attachment: five items for a secure style (e.g., “My workplace is like me”), five items for an avoidant style (e.g., “In my organization, I prefer to avoid certain places, even if that interferes with my work”), and five items for a preoccupied style (e.g., “Just thinking about my workplace makes me feel anxious”). Participants responded on a 7-point Likert scale ranging from 1 (totally disagree) to 7 (totally agree). Cronbach’s alpha indices for the three dimensions were .84, .71 and .86 respectively. Organizational citizenship behaviors were measured using the French version (Paillé, 2009) of Podsakoff et al.’s (1990) scale, which has 13 items measuring the following dimensions: helping behavior, altruism, civic virtue, and sportsmanship (e.g., “Acts as a peacemaker when others in the company have disagreements”). Items are rated on a 7-point Likert scale ranging from 1 (totally disagree) to 7 (Totally agree). In the present study, internal consistency was α = .67.

### Results

#### Preliminary Analysis

Before testing the hypotheses, given the cross-sectional nature of the study design, we checked whether the data were influenced by common-method bias (MacKenzie & Podsakoff, 2012) by comparing three alternative models. The first was a correlated three-factor model (OCB, political skills, and workplace attachment style) in which each item loaded on its latent factor. The second was composed of the set of items on the OCB scales and political skills inventory that loaded on the same latent factor, and the items of the workplace attachment style questionnaire that loaded on another latent factor. The third was a unifactorial model in which all the items of the three scales loaded on a single latent factor. The analyses were conducted with AMOS 20, using the maximum likelihood estimation method. The following fit indices were calculated to test the fit of the models: Carmines-McIver index, the ratio between χ^2^ and degrees of freedom, which must be between 2 and 3 to indicate a good fit of the model (McIver & Carmines, 1981); the Comparative Fit Index (CFI) and Non-Normed Fit Index (NNFI), with indices above .90 considered satisfactory (Hoyle, 1995); the Standardized Root Mean Square Residual (SRMR), with a value lower than .09 indicating satisfactory fit of the model to the data (Hu & Bentler, 1998). The models were compared using Δχ^2^. Table 1 shows the results of the analyses. The only model that shows all the satisfactory fit indices is the one with three related latent factors, and the Delta χ^2^ test indicates that this is the best model. This result suggests that our data are not subject to common-method bias.

**Table 1:** Measurement Model

Model	χ^2^	*df*	*p*	χ^2^/*df*	CFI	NNFI	SRMR	Δχ^2^	Δ*df*	*p*
M1 - One-factor	197	39	<.001	5.05	.73	.62	.11			
M2 - Correlated 2-factor	85	36	<.001	2.40	.91	.87	.09	112	3	<.001
M3 - Correlated 3-factor	67	33	<.001	2.05	.94	.90	.08	18	3	<.001

### Descriptive Statistics

Table 2 shows means, *SD*s and the bivariate correlations of the variables under study. In line with the literature, political skills were positively correlated with secure workplace attachment (*r* = .40, *p* < .01), and with OCB (*r* = .48, *p* < .01). Secure workplace attachment was positively correlated with OCB (*r* = .24, *p* < .01), whereas avoidant attachment was negatively correlated with OCB (*r* = -.16, *p* < .01). Finally, preoccupied workplace attachment was negatively correlated with OCB (*r* = -.41, *p* < .01).

**Table 2:** Means, Standard Deviations and Zero-Order Correlations (Alpha on the Diagonal)

Variable		*M*	*SD*	1	2	3	4	5	6	7	8	9
1	Gender (1 = F, 2 = M)			1								
2	Age	35.07	9.34	-.05	1							
3	Professional level			-.03	.12	1						
4	Organizational tenure	5.46	4.94	-.15*	.54**	.09	1					
5	Political skills	5.35	0.91	-.03	-.07	.08	-.23**	(.89)				
6	Secure workplace attachment	4.41	1.42	.15*	-.04	-.11	-.08	.40**	(.84)			
7	Avoidant workplace attachment	3.47	1.33	.05	-.01	-.27**	.01	.11	-.06	(.71)		
8	Preoccupied workplace attachment	2.59	1.42	.00	-.03	-.26**	.09	-.14	-.18*	.57**	(.86)	
9	Organizational citizenship behaviors	5.04	0.79	.00	.12	.09	-.05	.48**	.24**	-.16*	-.41**	(.67)

*Note*. *N* = 185.

*p < .05. **p < .01.

### Hypothesis Testing

To test the hypotheses, we analyzed three moderation models (one for each attachment style) using the PROCESS Macro for SPSS (Hayes, 2013). The contribution of moderation effects was tested using the *F* test on *R*_Change_, and we used simple slope analysis to interpret these effects. Several studies (Boswell et al., 2009; Moynihan & Pandey, 2007; Ng & Feldman, 2010) have shown the impact of organizational tenure on OCBs. For example, Moynihan and Pandey (2007) found that newly hired employees tend to immediately activate OCBs. However, these behaviors tend to decrease over time (Boswell et al., 2009; Ng & Feldman, 2010). Indeed, Kim (2018) demonstrated a negative relationship between organizational tenure and OCB. For these reasons, in our models we included organizational tenure as a covariate with the aim of neutralizing its effect. Our first model tested the relationship between political skills and OCBs (H_1_) and the moderation effect of secure workplace attachment (H_2_). Table 3 shows the results of moderation analysis. Concerning our first hypothesis, political skills were positively associated with OCBs (*B* = .46, *p* < .001, 95% CI [.32, .61]).

**Table 3:** Moderation Analysis of Secure Workplace Attachment on the Relation Between Political Skills and OCB

Variable	*B*	*SE*	95% CI
Constant	-.08	.07	[-.21, .06]
Political skills (PS)	.46***	.07	[.32, .61]
Secure workplace attachment (SWA)	.03	.07	[-.11, .17]
PS x SWA	.20**	.07	[.05, .35]
*Covariates*			
Age	.14	.08	[-.01, .29]
Gender	.06	.07	[-.08, .19]
Professional level	.08	.07	[-.05, .21]
Organizational tenure	.00	.08	[-.16, .16]
*R* ^2^	.29		
*F*	10.54**		

*Note*. *N* = 185. CI = Bootstrapping Confidence Interval. *R*^2^_change_ = .03. *F*_change_ = 7.23. *p* < .01.

*p < .05. **p < .01. ***p < .001.

**Figure 2:**
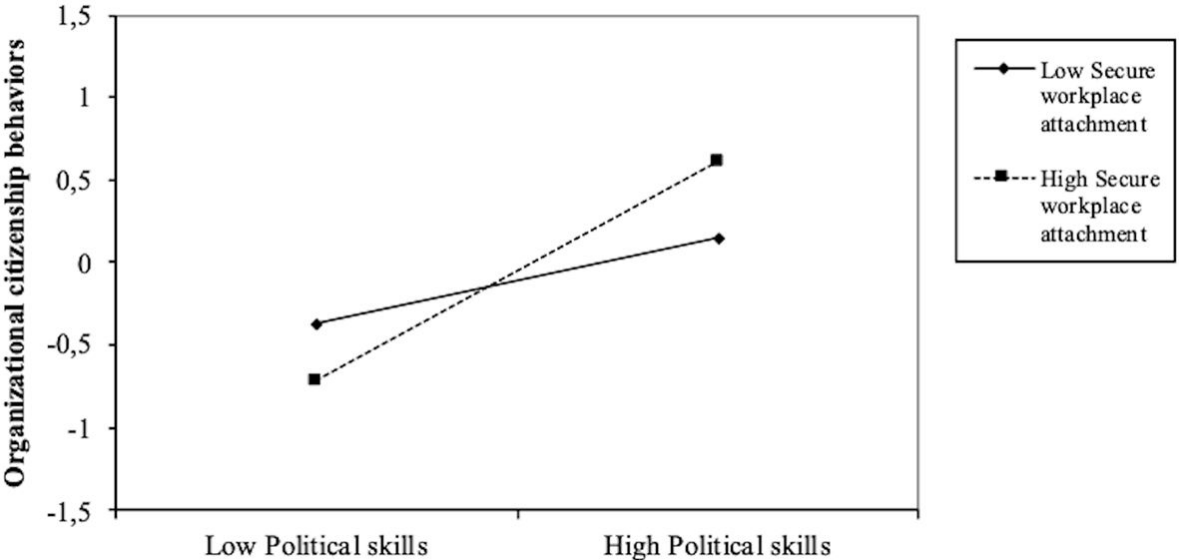
Simple Slope Analysis of the Moderation Effect of Secure Workplace Attachment on the Relation Between Political Skills and OCB

Our second hypothesis that secure workplace attachment would moderate the relationship between political skills and OCBs was confirmed (*B* = .20, *p* < .01, 95% CI [.05, .35]). Table 4 shows the conditional effects of the focal predictor at three moderator levels (-1SD, Mean, 1SD). Secure workplace attachment moderates the relationship between political skills and OCBs at all three levels. As shown in Figure 2, the interaction between secure attachment style and political skills significantly increased OCB levels.

**Table 4:** Conditional Effect of Focal Predictor

SWA	Effect	*SE*	*t*	*p*	LL 95% CI	UL 95% CI
-1 SD	.26	.10	2.52	<.05	.06	.47
0	.46	.07	6.37	<.001	.32	.61
+ 1 SD	.66	.10	6.41	<.001	.46	.87

*Note*. CI = confidence interval. LL = lower limit, UL = upper limit.

Our second model tested the moderating effect of avoidant workplace attachment on the relationship between political skills and OCBs *(H_3_*). Our third hypothesis is not confirmed. Table 5 shows a significant effect of political skills, *B* = .53, *p* < .001, 95% CI [.40, .66] and avoidant workplace attachment, *B* = -.24, *p* < .001, 95% CI [-.37, -.10] on OCBs, but the interaction effect was not significant, *B* = .00, *p* = n.s., 95% CI [-.15, .15].

**Table 5:** Moderation Analysis of Avoidant Workplace Attachment on the Relation Between Political Skills and OCB

Variable	*B*	*SE*	95% CI
Constant	.00	.06	[-.12, .13]
Political skills (PS)	.53***	.07	[.40, .66]
Avoidant workplace attachment (AWA)	-.24***	.07	[-.37, -.10]
PS x AWA	.00	.07	[-.15, .15]
*Covariates*			
Age	.15*	.07	[.00, .30]
Gender	.03	.06	[-.09, .15]
Professional level	-.04	.07	[-.17, .09]
Organizational tenure	.00	.08	[-.15, .16]
*R* ^2^	.31		
*F*	11.43***		

*Note*. *N* = 185. CI = Bootstrapping Confidence Interval. *R*^2^_change_ = .00, *F*_change_ = .00, *p* = n.s.

*p < .05. **p < .01. ***p < .001.

**Figure 3:**
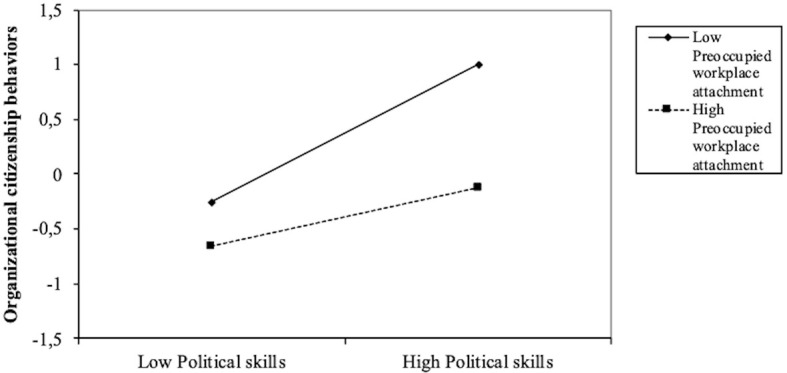
Simple Slope Analysis of the Moderation Effect of Preoccupied Workplace Attachment on the Relation Between Political Skills and OCB

Our final hypothesis that preoccupied workplace attachment would moderate the relationship between political skills and OCBs was confirmed. Table 6 shows a significant effect of political skills (*B* = .45, *p* < .001, 95% CI [.33, .57]) and preoccupied workplace attachment (*B* = -.38, *p* < .001, 95% CI [-.49, -.26]) on OCBs. The interaction variable is also significant (*B* = -.18, *p* < .01, 95% CI [-.30, -.06]). The conditional effect of the focal predictor (Table 7) indicates that the moderation effect of preoccupied workplace attachment is significant at all three moderator levels, and simple slope analysis (Figure 3) shows that the interaction between low levels of preoccupied workplace attachment style and high level of political skills significantly increased the level of OCBs.

**Table 6:** Moderation Analysis of Preoccupied Workplace Attachment on the Relation Between Political Skills and OCB

Variable	*B*	*SE*	95% CI
Constant	-.01	.06	[-.13, .10]
Political skills (PS)	.45***	.06	[.33, .57]
Preoccupied workplace attachment (PWA)	-.38***	.06	[-.49, -.26]
PS x PWA	-.18**	.06	[.30, -.06]
*Covariates*			
Age	.14*	.07	[.00, .28]
Gender	.03	.06	[-.08, .14]
Professional level	-.06	.06	[-.18, .06]
Organizational tenure	.02	.07	[-.12, .17
*R* ^2^	.41		
*F*	17.69***		

*Note*. *N* = 185. CI = Bootstrapping Confidence Interval. *R*^2^_change_ = .03. *F*_change_ = 8.83. *p* < .01.

*p < .05. **p < .01. ***p < .001.

**Table 7:** Conditional Effect of Focal Predictor

PWA	Effect	*SE*	*t*	*p*	LL 95% CI	UL 95% CI
-1 SD	.63	.08	7.57	<.001	.47	.80
0	.45	.06	7.45	<.001	.33	.57
+1 SD	.28	.09	3.00	<.01	.00	.44

*Note*. CI = confidence interval. LL = lower limit, UL = upper limit.

## Discussion

This study is part of a research field investigating how the way individuals relate to a specific environment impacts their behavior; for example, pro-environmental behavior (Song & Soopramanien, 2019), behavioral loyalty (Plunkett et al., 2019), and coping behavior (Ariccio et al., 2020). In this study, we tested whether workplace attachment style moderates the relationship between political skills and organizational citizenship behavior. In sum, political skills have an impact on organizational citizenship behaviors (OCB), and workplace attachment style moderates the relationship between political skills and OCB. More specifically, our first hypothesis that political skills are positively associated with OCBs was validated. Politically skilled individuals develop a sense of control over both others and their working environment (Ferris et al., 2007). This result is in line with the findings of Munyon and colleagues (2015) in a meta-analysis of 17 studies involving more than 3,500 participants. It confirms the role of political skills in the implementation of organizational citizenship behaviors (Perrewé et al., 2000), highlighting in particular their importance in people-oriented behaviors. Our second hypothesis that a secure attachment style to the workplace moderates the relationship between political skills and OCB was also confirmed. According to Bakker et al. (2005), the physical aspects of the workplace can be considered as job resources that stimulate personal growth. Employees develop a secure attachment style when the workplace is able to meet their needs. This job resource can therefore interact with political skills, seen as a personal resource that activates OCB. While secure attachment to the workplace does not in itself impact OCB, its interaction with political skills increases prosocial behavior at work. The third hypothesis postulated that avoidant workplace attachment would moderate the relationship between political skills and OCB. This hypothesis was not confirmed. However, we can see that an avoidant attachment style is characterized by a positive view of self and a negative view of place (Scannell, 2013; Scrima et al. 2017). Avoidant employees therefore think they are completely autonomous and that the place does not deserve them. This will make them less inclined to help a colleague. The ability to create social relations at work does not interact with this dimension of attachment. Regarding our fourth hypothesis that preoccupied workplace attachment would moderate the relationship between political skills and OCBs, our results reveal that individuals with a preoccupied attachment style and high levels of anxiety, even if they report strong political skills, do not show OCB. Here, the fear of rejection (Scrima, 2015) transcends the ability to understand relationships at work. It should be noted that the scores on this dimension are particularly low compared to the other two sub-constructs of place attachment. Finally, it is noteworthy that we found a negative and significant correlation between organizational tenure and political skills in our sample. However, this relationship disappeared in our tested models, demonstrating a spurious relationship between organizational tenure and political skills. This result is in line with those of Crawford et al. (2019) and Blickle et al. (2020) who found negative and non-significant relationships between these variables.

### Theoretical and Practical Implications

Our results open up new perspectives and avenues for future research. From a theoretical standpoint, they show how the impact of political skills (here on OCBs) cannot be understood without dissociating them from the physical characteristics of the workplace in which relationships are formed, and especially from the individuals’ relationship with the workplace. Many authors have advanced the hypothesis that any organization is a political arena (Crozier & Friedberg, 1977; Mintzberg, 1985). It is thus also important to understand the nature of the individuals’ relationship with this “political arena”. The workplace is seen here as a cognitive framework for relationships between individuals. Studies on workplace attachment style have shed light on the dynamics of multiple organizational behaviors (Ferris et al., 2007) that enable individuals to cope with stress (burnout, professional exhaustion). Within the theoretical framework of the JD-R model, our results indicate that workplace attachment styles may be the result of the ability of the physical characteristics of the workplace to meet employees’ needs. They also provide a further demonstration that the interaction between personal characteristics and work resources can impact the behavior of workers. This study also has practical implications. By providing a clearer understanding of the interplay between political skills and different forms of attachment to the workplace, it highlights the importance for organizations to identify and develop the political skills of their employees through training programs and tutoring (Ferris et al., 2008). At the same time, managers should take a holistic and systemic approach to their employees’ workplace attachment style. Furthermore, human resource management should consider the impact of the physical characteristics of the workplace on the behavior of individuals; good human resource management also involves ensuring that the workplace is able to meet the needs of employees. The strength and type of attachment to the workplace could provide useful information for human resource (HR) managers (Scrima et al., 2017), in order to set up programs to enable employees to develop more secure workplace attachment styles. This could be achieved through small group activities to raise employees’ awareness of their internal working models (IWM).

### Limitations

This research has several limitations. First, the sample size. The study was carried out with a single and relatively small sample (185 employees). It therefore seems essential to replicate it to confirm or qualify the results. Furthermore, it only involved organizations operating in France, and the possible impact of national employment policies on behaviors and attitudes towards work, in particular on OCBs, cannot be overlooked. It would also have been preferable to control further the type of workplace; while the participants had to be employed, have worked full-time for at least a year in the same organization and have a permanent contract, it is possible that they had more than one workplace with different characteristics. Another limitation concerns the cross-sectional design of the study, preventing the attribution of a cause and effect relationship between variables. The study was based on self-report questionnaires, which carries the risk of common-method bias (MacKenzie & Podsakoff, 2012). Although we took measures to reduce the impact of this bias on the data, the results should be interpreted with caution. Another limitation concerns the use of a global measure of political skills and OCBs. Studies (Ferris et al., 2005) have identified four dimensions of political skills: social astuteness, interpersonal influence, networking ability, and apparent sincerity. For OCBs, the literature describes five dimensions divided into two categories: behavior directed towards the individual (OCB-I), and behavior directed towards the organization (OCB-O) (Williams & Anderson, 1991). OCB-I includes altruism, courtesy, peacekeeping, and cheerleading, while OCB-O includes conscientiousness, civic virtue, and sportsmanship. The exploratory nature of our research did not allow us to develop more precise hypotheses of interactions between the sub-dimensions of political skills, the different workplace attachment styles and the different dimensions and categories of OCB. Finally, in this study, as suggested by Scrima (2020), the three attachment styles were used as separate dimensions. However, to align with the more recent literature on the dimensional measure of adult attachment style (Fraley et al., 2000), future research will be oriented to demonstrate whether the two dimensions of the WASQ, measuring the two styles of insecure attachment (preoccupied and avoidant) are able to combine to identify the four classic types of attachment (secure, worried, avoidant and disorganized).

### Conclusion

The aim of this research was to examine the moderating role of workplace attachment style on the relationship between political skills and organizational citizenship behaviors. Its results are consistent with the literature (Ferris et al., 2007; Scrima et al., 2017) while enriching and opening up new avenues for research. As the use of political skills and the adoption of OCBs depend on the extent to which the organization promotes this type of behavior, it would be interesting to analyze in more detail the moderating role of attachment styles in the workplace by varying the types of work environments and / or types of management. From a practical point of view, the results suggest that the quality of attachment to the workplace should be taken into account in order to develop the relationship between political skills and organizational citizenship behaviors and thus stimulate the behaviors that are important for the organization. Although these results need to be compared with those of other studies, we believe that they can be used by managers to improve the capacity of the work environment to meet the needs of their employees and hence their relationship with their work environment by promoting OCB.

## Appendix

**Workplace Attachment Style Questionnaire**

The following 15 sentences describe the mood that you might experience in your workplace. Please think about your workplace, its rooms and corridors, the color of its walls, its sounds, noises and smells and the people with whom you usually share these places. For each statement, place a cross on the scale to indicate your degree of agreement.

**Avoidant Workplace Attachment**
01_In my organization, I prefer to avoid certain places, even if that interferes with my work.
02_Nothing would make me stay at my workplace longer than necessary.
03_I dread going back to my workplace after a holiday.
04_I prefer not to go to certain places in my organization.
05_I tend to put off going to my workplace.

**Secure Workplace Attachment**
06_I’m attached to my workplace.
07_I would find it very difficult to leave my workplace for good.
08_My workplace is like me.
09_I enjoy the time that I spend in my workplace.
10_I wouldn’t enjoy working in another place as much.

**Preoccupied Workplace Attachment**
11_I often feel anxious in my workplace.
12_Just thinking about my workplace makes me feel anxious.
13_I find it difficult to feel at ease at my workplace.
14_Some places in my organization bring back bad memories.
15_I sometimes feel oppressed by my workplace.

Response type: 7-point Likert scale from “Totally disagree” to “Totally agree”
